# Genetic Determinants of Virulence between Two Foot-and-Mouth Disease Virus Isolates Which Caused Outbreaks of Differing Severity

**DOI:** 10.1128/mSphere.00294-19

**Published:** 2019-08-14

**Authors:** Tatsuya Nishi, Kazuki Morioka, Nobuko Saito, Makoto Yamakawa, Toru Kanno, Katsuhiko Fukai

**Affiliations:** a Exotic Disease Research Station, National Institute of Animal Health, National Agriculture and Food Research Organization, Tokyo, Japan; University of Maryland, College Park

**Keywords:** foot-and-mouth disease virus, genetic determinants, virulence

## Abstract

Individual foot-and-mouth disease virus (FMDV) strains reveal different degrees of infectivity and pathogenicity in host animals. The differences in severity among outbreaks might be ascribable to these differences in infectivity among FMDV strains. To investigate the molecular mechanisms underlying these differences, we estimated the infectivity of O/JPN/2000 and O/JPN/2010, which caused outbreaks of markedly different scales, in cell lines, Holstein cattle, and suckling mice. Viral growth of the two strains in cells was not remarkably different; however, O/JPN/2000 showed apparently low transmissibility in cattle. Mortality rates of suckling mice inoculated intraperitoneally with a 50% tissue culture infective dose (TCID_50_) of 10 for O/JPN/2000 and O/JPN/2010 also differed, at 0% and 100%, respectively. To identify genes responsible for this difference in infectivity, genetic regions of the full-length cDNA of O/JPN/2010 were replaced with corresponding fragments of O/JPN/2000. A total of eight recombinant viruses were successfully recovered, and suckling mice were intraperitoneally inoculated. Strikingly, recombinants having either VP1 or 3D derived from O/JPN/2000 showed 0% mortality in suckling mice, whereas other recombinants showed 100% mortality. This finding indicates that VP1, the outermost component of the virus particle, and 3D, an RNA-dependent RNA polymerase, are individually involved in the virulence of O/JPN/2010. Three-dimensional structural analysis of VP1 confirmed that amino acid differences between the two strains were located mainly at the domain interacting with the cellular receptor. On the other hand, measurement of their mutation frequencies demonstrated that O/JPN/2000 had higher replication fidelity than O/JPN/2010.

**IMPORTANCE** Efforts to understand the universal mechanism of foot-and-mouth disease virus (FMDV) infection may be aided by knowledge of the molecular mechanisms which underlie differences in virulence beyond multiple topotypes and serotypes of FMDV. Here, we demonstrated independent genetic determinants of two FMDV isolates which have different transmissibility in cattle, namely, VP1 and 3D protein. Findings suggested that the selectivity of VP1 for host cell receptors and replication fidelity during replication were important individual factors in the induction of differences in virulence in the host as well as in the severity of outbreaks in the field. These findings will aid the development of safe live vaccines and antivirals which obstruct viral infection in natural hosts.

## Abstract (precis)

Efforts to understand the universal mechanism of foot-and-mouth disease virus (FMDV) infection may be aided by knowledge of the molecular mechanisms which underlie differences in virulence beyond multiple topotypes and serotypes of FMDV. Here, we demonstrated independent genetic determinants of two FMDV isolates which have different transmissibility in cattle, namely, VP1 and 3D protein. Findings suggested that the selectivity of VP1 for host cell receptors and replication fidelity during replication were important individual factors in the induction of differences in virulence in the host as well as in the severity of outbreaks in the field. These findings will aid the development of safe live vaccines and antivirals which obstruct viral infection in natural hosts.

## INTRODUCTION

Foot-and-mouth disease virus (FMDV) is classified in the genus *Aphthovirus* of the family *Picornaviridae*. Its genome is composed of a single-stranded positive-sense RNA of approximately 8.4 kb in length which is divided into an S fragment and L fragment by poly(C) sequence at the 5′ terminus of the genome. The open-reading frame of FMDV is composed of 12 proteins, L, VP1 to VP4, 2A, 2B, 2C, 3A, 3B, 3C, and 3D. The FMDV capsid surface is covered by VP1, -2, and -3 and held by VP4, which is buried within the virion (1).

Foot-and-mouth disease (FMD) is the most contagious disease of mammals and causes severe economic damage to livestock industries. FMDV-infected animals show vesicles on the mouth and nostrils and around the breasts and feet as typical lesions. The virulence and infectivity of FMDV in cattle or swine is strain dependent. In 1997, for example, FMDV with atypical virulence which showed high morbidity and mortality in swine but did not affect cattle was confirmed and shown to have caused a devastating outbreak in Taiwan (2). In contrast, FMDVs which revealed limited virulence in cattle were isolated in South Korea and Argentina (3, 4). Our previous and several other studies have reported that virulence in hosts and virus growth in cell culture were related and that the genes responsible were two substitutions on the 133rd amino acid in VP2 and the 56th amino acid in VP3, a deletion of L^pro^, part of a deletion in 3A, and internal ribosome entry site (IRES)-3′ untranslated region (UTR) modulation (5–9). To date, however, few studies have described the genes responsible for FMDV pathogenicity among multiple topotypes.

Japan has experienced two FMD outbreaks over the last 100 years. These notably differed in severity; however, the 2000 outbreak was limited to four cattle farms, whereas the 2010 outbreak spread to 292 farms and resulted in the slaughter of approximately 300,000 animals (10, 11). This differing severity was suspected to be due to differences in pathogenicity in cattle in the field. In 2000, infected cattle showed only fever, salivation, and erosion, and vesicular development, the typical clinical sign, was not confirmed. In 2010, in contrast, the typical clinical signs of fever, salivation, ulcers in the mouth, and vesicular development were all confirmed. While this difference in pathogenicity might have caused the difference in the severity of the two outbreaks, the molecular mechanisms underlying the pathogenicity of the virus are not well understood.

In this study, we compared the infectivity of two virus strains isolated from these outbreaks and having different topotypes, O/JPN/2000 and O/JPN/2010, in cattle (12, 13). We also compared their viral growth in cell culture and virulence in suckling mice. In addition, the genes responsible for the difference in infectivity were evaluated using genetic recombinants between the two strains.

## RESULTS

### Experimental infection with O/JPN/2000 in cattle.

**FIG 1:**
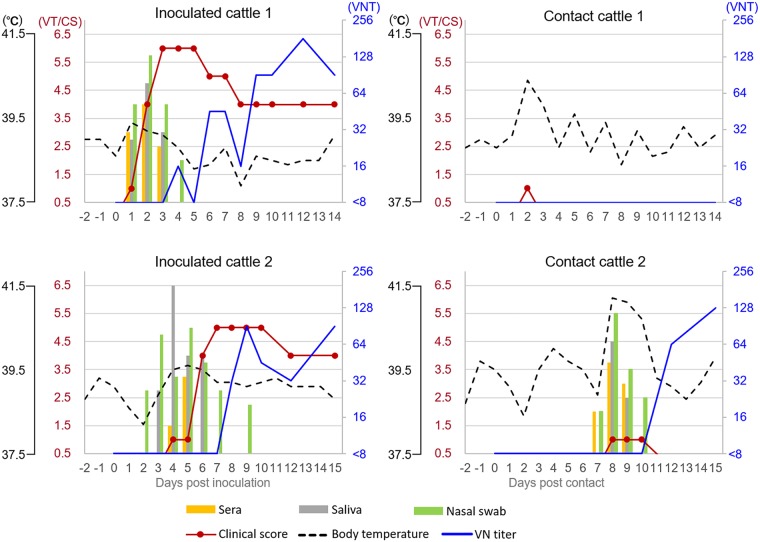
Time course of infection in cattle inoculated with the FMDV O/JPN/2000 isolate or kept in contact with inoculated cattle. Times on the *x* axes are given in dpi and dpc. Viral titers (VT; as log_10_ TCID_50_/0.1 ml) in sera and mouth and nasal swabs are shown together with the development of clinical signs (CS; score of 0 to 6) and rectal temperature (°C) on the left *y* axes; antibody titers measured in VNTs are on the right *y* axes.

In inoculated cattle 1 and 2, vesicular lesions were initially found at the injection site on the tongue at 1 and 4 days postinfection (dpi), and new lesions began to develop on the feet at 2 and 6 dpi, respectively (Table 1). Lesions on the feet were found at the hind limbs from 2 or 6 dpi, and confirmed at all four limbs on 3 or 7 dpi, respectively. Lameness and/or excess salivation was observed from 1 or 4 dpi, respectively; however, pyrexia was not confirmed. Total clinical scores reached 6 and 5, respectively. From these cattle, virus was isolated from the sera (1 to 3 or 4 to 5 dpi), saliva (1 to 6 dpi), and nasal swabs (2 to 3 dpi) using LFPK-αvβ6 cells. Antibodies were detected by virus neutralization test (VNT) from 6 or 8 dpi (Fig. 1).

**TABLE 1:** Clinical scores in cattle infected with FMDV O/JPN/2000 or O/JPN/2010

Inoculated virus	Clinical sign	Inoculated no. 1	Contact no. 1	Inoculated no. 2	Contact no. 2
O/JPN/2000	Pyrexia	ND^*a*^	ND	ND	8
	Excess salivation	1^*b*^	ND	4	ND
	Vesicular development				
	Tongue	1	ND	4	ND
	Fore limb				
	Right	3	ND	7	ND
	Left	3	ND	6	ND
	Hind limb				
	Right	2	ND	6	ND
	Left	2	ND	6	ND
O/JPN/2010^*c*^	Pyrexia	ND	5	3	4
	Excess salivation	3	4	2	6
	Vesicular development				
	Tongue	1	5	1	6
	Fore limb				
	Right	5	5	3	6
	Left	6	5	4	6
	Hind limb				
	Right	3	5	3	6
	Left	2	5	4	6

a ND, not detected.

b The postinoculation or postcontact day on which clinical signs were initially observed.

c Results of clinical signs on infection with O/JPN/2010 in this table have been published elsewhere (15).

On the other hand, in contact cattle 1 and 2, which were placed in close proximity to the inoculated cattle, although fever was confirmed on 2 and 8 dpi, respectively, no vesicular lesions were confirmed (Table 1). Clinical scores were therefore limited to 1. Viruses were isolated from the sera (7 to 9 days postcontact [dpc]), saliva (8 to 9 dpc), and nasal swabs (7 to 10 dpc) of the contact cattle 2. Antibodies were detected by VNT from 11 dpc (Fig. 1). These data indicate that it took 5 days to transmit FMD from inoculated cattle 2 to contact cattle 2.

### Comparison of viral growth of O/JPN/2000 and O/JPN/2010 in cells.

**FIG 2:**
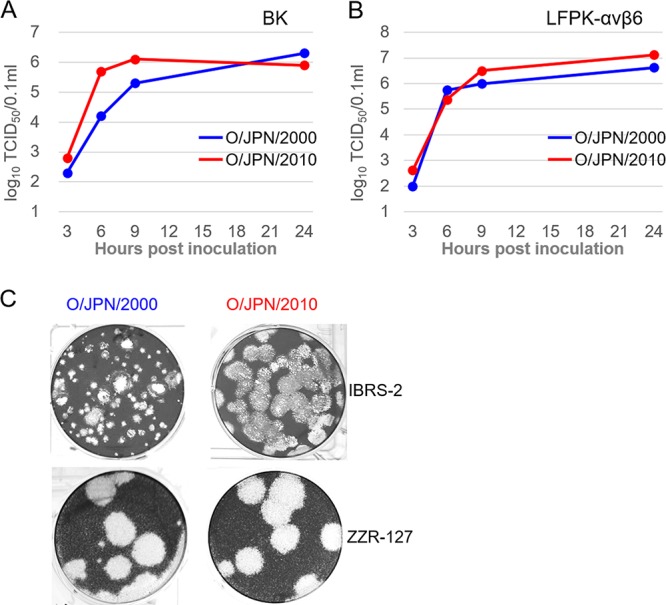
Growth characteristics of O/JPN/2000 and O/JPN/2010. One-step growth curves in BK (A) and LFPK-αvβ6 (B) cells. The cell monolayers were inoculated with each virus at an MOI of 0.1 and incubated at 37°C. Samples of supernatants were collected at the indicated times and viral infectivity was determined. (C) Comparison of the plaque sizes of O/JPN/2000 and O/JPN/2010. IBRS-2 or ZZR-127 monolayer cells were inoculated with the two strains. The cultures were fixed 1 day after the inoculation and stained with crystal violet.

Bovine kidney (BK) and LFPK-αvβ6 cell monolayers were inoculated with O/JPN/2000 and O/JPN/2010. These were harvested at subsequent time points and the amount of virus present was determined (Fig. 2A and B). Growth curves of both viruses reached a plateau at 6 or 9 h postinoculation. Virus titers reached 10^6.1^ or 10^6.3^ 50% tissue culture infective dose (TCID_50_)/0.1 ml at maximum in BK cells, and 10^6.6^ or 10^7.1^ TCID_50_/0.1 ml in LFPK-αvβ6 cells, respectively. Although O/JPN/2010 showed better growth, we did not see a remarkable difference between the strains. Plaques of O/JPN/2000 and O/JPN/2010 on IBRS-2 and ZZR-127 cell monolayers were visualized by staining with crystal violet (Fig. 2C). Even though O/JPN/2000 showed a few smaller plaques in IBRS-2, it was confirmed that there was no remarkable difference between the strains.

### Comparison of amino acid sequences of O/JPN/2000 and O/JPN/2010.

Genome sequences of O/JPN/2000 and O/JPN/2010 were aligned, and the predicted amino acid sequences in each viral protein were compared. Among the 12 proteins which compose the open-reading frame of FMDV, only VP4 showed no difference between the two strains. A total of 106 amino acid differences were confirmed across the viral genome (Table 2).

**TABLE 2:** Comparison of amino acid sequences of O/JPN/2000 and O/JPN/2010

Genome region	Amino acid length	No. of differences
L^pro^	201	24
VP4	85	0
VP2	218	10
VP3	220	11
VP1	213	17
2A	16	1
2B	154	3
2C	318	9
3A	153	9
3B	71	6
3C^pro^	213	5
3D^pol^	471	11
		
Total	2,333	106

### Construction of recombinant FMDV using the two strains.

**FIG 3:**
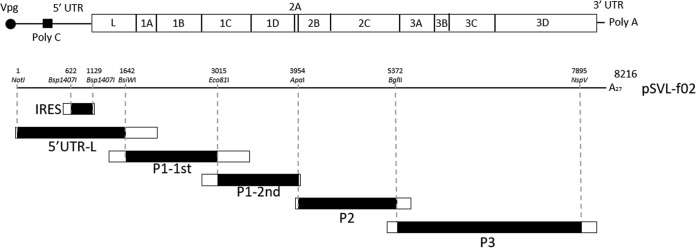
Construction of recombinant FMDV using O/JPN/2000 and O/JPN/2010. Each insertion gene of O/JPN/2000 amplified by PCR was ligated with the full-length infectious cDNA of O/JPN/2010 (pSVL-f02) by using appropriate restriction enzymes.

Infectious cDNA of O/JPN/2010 was comprehensively recombined to the corresponding positions of O/JPN/2000 (Fig. 3, Table 3). Each recombinant plasmid was transfected into Cos-7 cells and passaged in ZZR-127 cells. A total of eight recombinant viruses, with recombined 5′ UTR, IRES, and the first half and the second halves of P1, VP1, P2, 3A-B, and 3D regions between the two strains, showed cytopathic effect (CPE) on ZZR-127 cells and were successfully passaged for subsequent studies. Genome sequences of all virus stocks of recombinants were confirmed. Although one nonsynonymous substitution on the 328th nucleotide (from Ser to Pro on the 110th amino acid) in 2B was confirmed in IRES/2000 vSVL-f02, any additional mutations were not observed.

**TABLE 3:** Oligonucleotide primers used for construction of chimeric recombinants

Recombinant	Type^*a*^	Sequence	Nucleotide position^*b*^
5′ UTR/2000 vSVL-f02	F in	GCAGGCGGCCGCTTGAAAGGGGGCGTTAGGGTCTC	NotI plus 1–23 (+)
	R in	TCCGTTGCGGGTAGTGAGGATGC	1993–2015 (−)
IRES/2000 vSVL-f02	F in	AACCACAAGATGAACCTTCACC	509–530 (+)
	R in	GTGTACAACAAAGCGATGAAACAGTC	1110–1135 (−)
P1-1st/2000 vSVL-f02	F in	CTTTCTTCGACTGGGTCTACCAC	1294–1316 (+)
	R in	GGTGTACGCGTAATCAGCCGCCG	3097–3119 (−)
P1-2nd/2000 vSVL-f02	F in	ACCAACTTCCTTGATGTGGCTGA	2733–2755 (+)
	R in	ACGTCAGAGAAGAAGAAGGGCCC	3954–3976 (−)
P2/2000 vSVL-f02	F in	CCAACCCTGGGCCCTTCTTCTTC	3946–3968 (+)
	R in	GCGGATCATGATCACTATGTTTGCC	5579–5603 (−)
P3/2000 vSVL-f02	F in	TCAGTTTGGTACTGCCCACCTGA	4827–4849 (+)
	R in	ATTTTCACTCCTACGGTGTC	8139–8158 (−)
VP1/2000 vSVL-f02	F in	TCGGCAACAGACCACCTCCACAGGTGAGTCGGCTGA	3260–3295 (+)
	R in	CAGATCAAAGTTCAAAAGCTGTTTCACAGGCGCCA	3886–3920 (−)
	F vec	TTGAACTTTGATCTGCTCAAGTTGGCA	3906–3932 (+)
	R vec	GTGGTCTGTTGCCGAGCGTCCACAGGCA	3247–3274 (−)
3A-B/2000 vSVL-f02	F in	CAATTCCTTCCCAAAAGGCTGTACTGTA	5377–5404 (+)
	R in	GGGGGCACCACTCTCAGTGACAAT	6033–6056 (−)
	F vec	GAGAGTGGTGCCCCCCCGACCGA	6042–6064 (+)
	R vec	TTTGGGAAGGAATTGAGATCTGCTTGA	5365–5391 (−)
3C/2000 vSVL-f02	F in	TTGATCGTCACCGAGAGTGGT	6030–6050 (+)
	R in	CTCGTGGTGTGGTTCGGGGTCGATGTGT	6656–6683 (−)
	F vec	GAACCACACCACGAGGGGTTGATCGTA	6669–6695 (−)
	R vec	CTCGGTGACGATCAAGTTCCTAGCTTTCA	6016–6044 (−)
3D/2000 vSVL-f02	F in	GAACCACACCACGAGGGATTGATAGTTGACACCA	6669–6702 (+)
	R in	CTGAGAGATTATGCGTCACCGCACACGGCGTT	8073–8104 (−)
	F vec	CGCATAATCTCTCAGATGTCACAATTGGCAGA	8090–8121 (+)
	R vec	CTCGTGGTGTGGTTCAGGGTCGATGTGT	6656–6683 (−)

a F in, forward primer for insert gene; R vec, reverse primer for vector gene.

b Nucleotide position corresponds to the nucleotide sequence of O/JPN/2010 290-1E (LC036265).

### Pathogenicity of the parental viruses and recombinant FMDV in suckling mice.

Suckling mice were intraperitoneally inoculated with viruses, and their survival rates were observed for 7 days. The 50% lethal doses (LD_50_s) of O/JPN/2000 and vSVL-f02 were determined to be 10^2.2^ and 10^0.1^ TCID_50_, respectively, indicating that the recovered virus of infectious cDNA of O/JPN/2010 had higher pathogenicity in suckling mice. Based on this result, the mortality rates of suckling mice inoculated with 10 TCID_50_ of O/JPN/2000 and vSVL-f02 were 0% and 100%, respectively.

**FIG 4:**
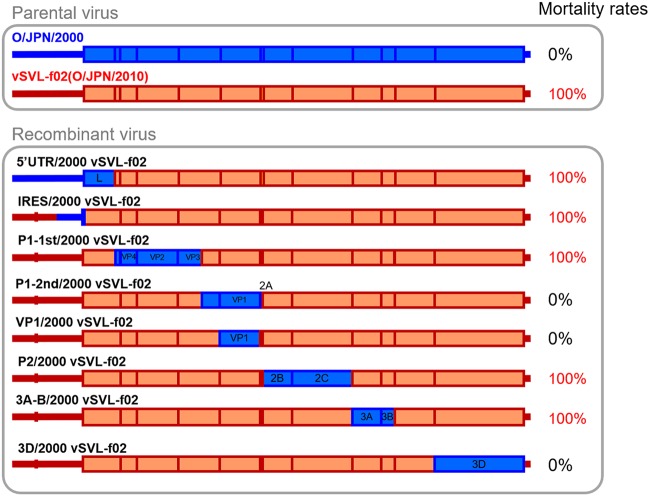
Schematic diagram and pathogenicity in suckling mice of recovered recombinant FMDVs between O/JPN/2000 and O/JPN/2010. Blue and red genes indicate genes from O/JPN/2000 and O/JPN/2010, respectively. Mortality rates of suckling mice inoculated with 10^1^ TCID_50_ of each recombinant virus are indicated on the right.

Suckling mice were also intraperitoneally inoculated with each recombinant virus at 10 TCID_50_, and their survival rates were observed. Chimeric viruses 5′ UTR/2000 vSVL-f02, IRES/2000 vSVL-f02, P1-1st/2000 vSVL-f02, P2/2000 vSVL-f02, and 3A-B/2000 vSVL-f02 showed 100% mortality, as with the virulent parental virus, vSVL-f02 (Fig. 4). This means that recombination of these genetic regions did not affect their pathogenicity. In contrast, mortality rates of suckling mice inoculated with 10 TCID_50_ of each of P1-2nd/2000 vSVL-f02, VP1/2000 vSVL-f02, and 3D/2000 were 0%. Therefore, VP1 and 3D proteins were individually suspected of being responsible for the pathogenicity of O/JPN/2010.

### Comparison of amino acid sequences and three-dimensional structures of VP1 and 3D.

**FIG 5:**
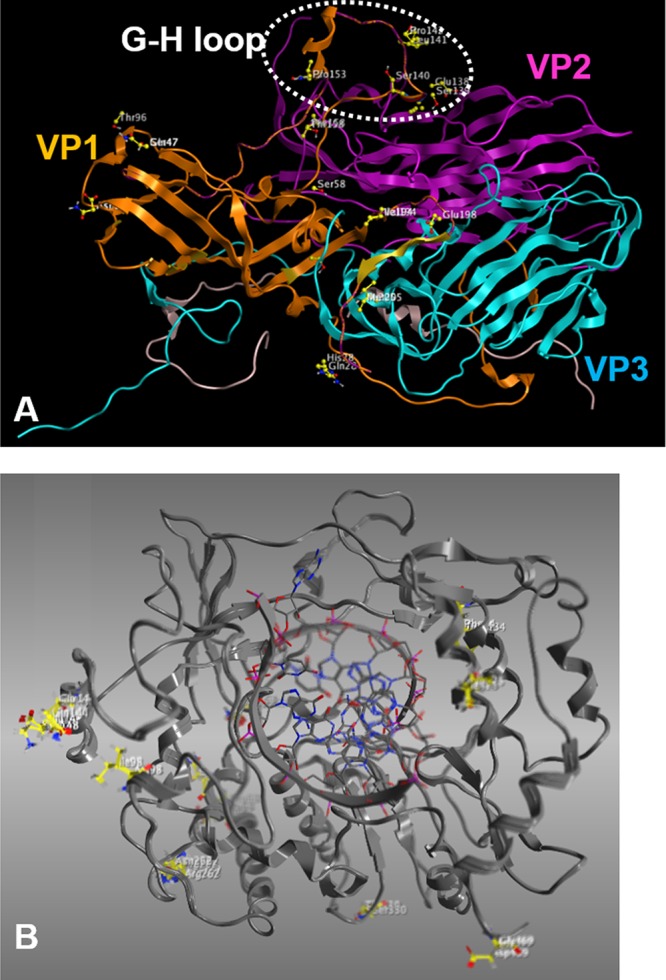
Positions of amino acid differences between O/JPN/2000 and O/JPN/2010 in three-dimensional structures. The amino acid differences between O/JPN/2000 and O/JPN/2010 were plotted as yellow dots on the three-dimensional structures of VP1 (A) and 3D (B) obtained from the Protein Data Bank (PDB; accession numbers 5NER and 4WZM, respectively) using MOE software.

Comparison of the VP1 amino acid sequences of O/JPN/2000 and O/JPN/2010 revealed 17 differences (Table 4). Locations of the amino acid differences in the three-dimensional structures of the structural proteins VP1, VP2, and VP3 were predicted using MOE software (Fig. 5A). Nine of these, including six consecutive amino acid differences, were confirmed near the RGD receptor binding domain.

**TABLE 4:** Seventeen amino acid differences between O/JPN/2000 and O/JPN/2010 in VP1

Strain	aa no.^*a*^											
	28	47	58	85	96	137–142	153	158	185	194	198	212
O/JPN/2000	Q	Q	**A**	N	T	**GESPVT**	**Q**	**T**	T	I	E	L
O/JPN/2010	H	S	**S**	D	A	**AGGSLP**	**P**	**P**	A	V	A	S

a Nine amino acid differences located on the G-H loop receptor binding domain are in boldface font. Amino acid numbers were annotated with the VP1 of O/JPN/2010-290/1E (GenBank LC036265).

In the 3D amino acid sequence, 11 differences were identified between the two strains (Table 5). We also analyzed the three-dimensional structure of 3D protein and plotted amino acid differences between the two strains (Fig. 5B). Based on a previous report about the structure of FMDV 3D polymerase (14), three amino acid differences (amino acid [aa] numbers [no.] 98, 144, and 148) were confirmed on the finger domain, three amino acid differences (aa no. 34, 330, and 425) were confirmed on the palm domain, and one amino acid difference (aa no. 469) was confirmed on the thumb domain.

**TABLE 5:** Eleven amino acid differences between O/JPN/2000 and O/JPN/2010 in 3D

Strain	aa no.^*a*^										
	34	63	68	98	144	148	254	262	330	425	469
O/JPN/2000	F	N	E	A	E	K	N	N	S	T	D
O/JPN/2010	Y	D	P	I	Q	E	S	R	T	I	G

a Amino acid numbers are annotated with the 3D of O/JPN/2010-290/1E (GenBank LC036265).

### Comparison of mutation frequencies of O/JPN/2000 and O/JPN/2010.

To determine the mutation frequencies of O/JPN/2000 and O/JPN/2010, 700-bp fragments of the structural protein-coding regions from 70 individual clones for each viral population were sequenced and the average numbers of mutations per 10^4^ nucleotides (nt) were calculated. The mutation frequencies of O/JPN/2000 and O/JPN/2010 were 5.21 and 7.98 mutation/10^4^ nucleotides, respectively.

## DISCUSSION

In this study, Holstein cattle which were intradermally inoculated with O/JPN/2000 unexpectedly showed vesicular development in all four limbs. However, only one of two contact cattle were infected 5 days after virus excretion from inoculated cattle and showed only mild clinical signs (Fig. 1, Table 1). In contrast, in our previous study (15), cattle inoculated with O/JPN/2010 and contact cattle all showed fever, salivation, lameness, and vesicular development. Furthermore, the contact cows were confirmed to be infected within only 2 days after virus excretion from the inoculated cattle. Moreover, clinical scores of both inoculated and contacted cattle reached 5 to 6. These data clearly demonstrate that O/JPN/2000 has very low transmissibility and pathogenicity to contact cattle.

Several studies using wild-type strains and their mutants have reported that virus growth in cell culture and virulence in hosts were closely related (5–9). For example, a mutant with a partial deletion in 3A protein did not replicate efficiently in bovine cells *in vitro* and was attenuated in cattle (7, 8). In the present study, in contrast, we saw no remarkable difference in viral growth in BK and LFPK-αvβ6 cells between O/JPN/2000 and O/JPN/2010 (Fig. 2). In unweaned mice, on the other hand, which have been widely used as a practical model of FMDV pathogenicity (16–18), O/JPN/2000 and O/JPN/2010 showed a definite difference in virulence (Fig. 4). Our data support the idea that mortality in infected suckling mice is an effective index for comparing the infectivity of FMDVs, particularly those which belong to different genetic topotypes.

As described above, infectious cDNA of O/JPN/2010 was comprehensively recombined to the corresponding positions of O/JPN/2000, since amino acid differences between the two strains were confirmed all over the genome (Fig. 3, Table 2). A total of eight recombinant viruses were recovered from transfected cells regardless of the number of amino acid differences in each recombined fragment (Fig. 4). Only a few viruses, such as hepatitis C virus, are known to be flexibly useful for comprehensive fragmental recombination to this extent. This genetic flexibility might be one reason for the enormous genetic variation in FMDV and allows the generation of recombinants in the field (19). On the other hand, recombinant virus of 3C protein was not recovered. 3C in FMDV plays a role as protease in the viral replication step. Although it has been reported to have “relaxed specificity” which discriminates only weakly in favor of P1-Gln over P1-Glu—in contrast to other proteases of picornavirus that strongly favor P1-Gln (20)—its adoptability might be restricted in combination with other genetic regions.

The pathogenicity of the parental viruses and recombinant FMDV in suckling mice indicated that VP1 and 3D proteins were individually responsible for the pathogenicity of O/JPN/2010 (Fig. 4). VP1 is the outermost component of the virus particle and is responsible for receptor binding (21, 22). Analysis of the three-dimensional structure of the viral protein showed that 9 of 17 amino acid differences between the two strains were located near the G-H loop (Fig. 5A, Table 4), indicating that the two strains have different selectivity or affinity to host cell receptors. Although one-step growth in the cell monolayers of the two strains was not remarkably different (Fig. 2), in fact, they showed significantly different viral features after serial passages in cells. Namely, O/JPN/2000 and O/JPN/2010 were serially passaged 10 times in BHK cells and suckling mice were inoculated (data not shown). In O/JPN/2000 virus stock at the primary stage, two types of viruses were observed: one shows small plaque and avirulent pathogenicity in suckling mice, whereas another shows large plaque and higher pathogenicity (5). As with our previous study, O/JPN/2000 after the passages showed two substitutions on the 133rd amino acid in VP2 and the 56th amino acid in VP3, which is known as a heparin sulfate binding site and which influences plaque size and pathogenicity in cattle (23), and significantly decreased mortality in suckling mice (LD_50_, >10^3.0^ TCID_50_). On the other hand, no nonsynonymous substitution or change in mortality in suckling mice was confirmed in serially passaged O/JPN/2010. These data also support the hypothesis that the two strains have different selectivity to host cell receptors.

This finding that the capsid coding sequences are determinants of FMDV pathogenicity is consistent with a previous study using interserotypically recombined chimeric viruses (24). VP1 has been reported to modulate host immune factors, such as inhibiting type I interferon response in cells by interacting with soluble resistance-related calcium binding protein (25). In addition, previous reports indicated that FMDV infection induces cell death by apoptosis mediated by interaction with the integrin receptor (26, 27). In our previous study, terminal deoxynucleotidyl transferase-mediated dUTP nick end labeling (TUNEL)-positive labeling in pigs inoculated with O/JPN/2000 was weaker than in pigs inoculated with O/JPN/2010 (28). This finding suggests that the viral function which induces apoptosis differs between O/JPN/2000 and O/JPN/2010. The programmed dead cells are processed into an apoptotic small body which is phagocytosed by macrophages. Although FMDV replication in macrophages has not been confirmed, the majority of macrophages carried infectious virus for 10 to 24 h. Such macrophages would play a role in the transport of infectious FMDV to different sites in the body, where it could be released to infect other cells for replication (29, 30). Therefore, the selectivity or affinity for receptors of the virus is probably related to its infectivity in cattle. Further study of these protein functions would help elucidate the mechanism of virulence of FMDV.

3D protein of FMDV performs as an RNA polymerase and has a right-hand structure composed of finger, palm, and thumb domains (14). According to a three-dimensional structure analysis of the protein, three, three, and one amino acid differences were found on the finger, palm, and thumb domains, respectively (Fig. 5B). In addition, 6 of 11 amino acid differences were confirmed in the region suggested to be responsible for protein-protein interaction (14). Among picornaviruses, relationships between the structure and function of coxsackievirus B3 polymerase have been reported (31). Mutations located at the top of the finger domain affect elongation rates, whereas mutations on the palm domain have the greatest effect on mutation frequencies. Interestingly, FMDV with low-fidelity polymerase is reported to be attenuated in the host (32–34). Namely, lower replication fidelity could induce restricted quasispecies diversity and affect the adaptability and virulence of the strain. In their study, mutants which showed 1.51- to 1.88-fold higher replication fidelity exhibited 10- to 100-fold lower virulence in suckling mice compared to those of the wild type (34). Using this method, the mutation frequencies (mutations per 10^4^ nt) of O/JPN/2000 and O/JPN/2010 in the P1 region (2,699 to 3,398 nt) were determined. O/JPN/2000 showed approximately 1.53-fold higher fidelity than O/JPN/2010. These data, and the 10^2.1^-fold lower virulence of this strain than O/JPN/2010 in suckling mice, demonstrate that replication fidelity is one factor which accounts for the adaptability and virulence of the virus in the host. Further study of the correlations between FMDV polymerase fidelity and virulence will aid the development of live attenuated FMDV vaccine candidates, as the enhanced replication fidelity promises high stability and safety.

VP1 and 3D sequences of O/JPN/2000 and O/JPN/2010 were aligned and compared with FMDV sequences available in GenBank. Among the 17 VP1 amino acid differences between the two strains, the six consecutive amino acid differences (aa no. 137 to 142) near the RGD receptor binding domain were specific sequences to each genetic lineage, namely ME-SA/PanAsia and SEA/Mya-98 lineages, respectively, though other amino acid sequences were common in serotype O strains. On the other hand, among the eleven 3D amino acids in Table 5, Asn of 63rd and 262nd aa and Ser of 330th aa of O/JPN/2000 were specifically confirmed in virus strains of ME-SA/PanAsia lineage, whereas those of O/JPN/2010 were common among multiple topotypes. In addition, Ala of the 98th aa was unique to the O/JPN/2000 strain among all FMDV strains in GenBank, although it is the same attribute with other amino acids confirmed at this position, valine and isoleucine (hydrophobic amino acids). Additional studies are needed to elucidate whether and how these amino acid motifs affect their protein function.

In the present study, we demonstrated that O/JPN/2000 and O/JPN/2010 had completely different transmissibility in inoculated cattle and virulence in suckling mice and that this difference was independently due to differences in VP1 and 3D protein. Selectivity of VP1 to receptors and replication fidelity of the polymerase are suspected to be key individual factors in the difference in infectivity and pathogenicity in the host.

## MATERIALS AND METHODS

### Cells and viruses.

Primary bovine kidney (BK), IBRS-2, BHK-21, Cos-7, and CPK cells were grown in Eagle’s minimum essential medium (MEM; Nissui Pharmaceutical, Tokyo, Japan). ZZR-127 (35) and LFPK-αvβ6 cells (36, 37) were grown in Dulbecco’s modified Eagle’s medium-nutrient mixture F-12 (DMEM; Life Technologies, Carlsbad, CA, USA) supplemented with fetal bovine serum. The cells were maintained at 37°C in a 5% CO_2_ atmosphere. Virus isolation was performed according to the 2017 OIE terrestrial manual (38). During the outbreak in 2000 and 2010, cells derived from both bovine kidney and porcine kidney were used for the isolation of FMDV from clinical samples because of their high susceptibilities. Cos-7 cells are efficiently transfected with pSVL and thus were used for DNA transfection. ZZR-127 cells were used to prepare stocks of chimeric viruses, because viral growth in this cell line is the highest. LFPK-αvβ6 cells were used for virus titration and neutralization test because of their high sensitivity to FMDV and efficient growth.

The virus strain O/JPN/2010-290/1E (GenBank LC036265) was isolated from epithelial tissue of cattle in Japan using CPK cells and passaged three times in CPK cells (39). O/JPN/2000 (GenBank AB079061/062) was isolated from oropharyngeal fluid material from cattle in Japan using BK cells (10) and passaged two times in BK cells and two times in LFPK-αvβ6 cells. As with our previous study (5), O/JPN/2000 after a few passages in BK or BHK cell lines shows small plaques and avirulent pathogenicity in suckling mice due to two substitutions on the 133rd amino acid in VP2 and the 56th amino acid in VP3. On the other hand, LFPK-αvβ6 cells stably express both the αv and β6 bovine integrin subunits, which is a principal receptor for FMDV in host cells. Therefore, in this study, viral stock of O/JPN/2000 strain was prepared using LFPK-αvβ6 cells to keep its infectivity. All stock viruses were stored at –80°C.

### Experimental infection of O/JPN/2000 in Holstein cows.

Two 6-month-old Holstein cows were inoculated subepidermolingually with 1 ml of 10^6^ TCID_50_ (titrated using IBRS-2 cells) of FMDV O/JPN/2000 as described previously (15). Two additional Holstein cattle of the same age were housed with them at 0 dpi. This experimental infection was performed in cubicles of approximately 14 m^2^ in a high-containment facility at the NIAH. The cubicles were kept at 25°C and provided 10 to 15 air changes per h during the experimental period. Clinical signs, virus excretion, and antibody responses of the infected animals were observed for approximately 2 weeks. Blood for serum production was collected from a cervical vein using a vacuum blood collection tube (Venoject II; Terumo Corp., Tokyo, Japan). Saliva was collected from the oral cavity using a roll-shaped synthetic saliva collector (Salivette; Sarstedt KK, Tokyo, Japan) and forceps. Nasal swabs were collected from the nasal cavity using a cotton swab (Men-tip; JCB Industry Ltd., Tokyo, Japan). Esophageal-pharyngeal fluid was collected using a probang cup. Collection of clinical samples except for the esophageal-pharyngeal fluid was performed daily until 10 days postinfection (dpi) and at 2-day intervals thereafter. The esophageal-pharyngeal fluids were collected at 0, 10, 12, and 14 or 15 dpi. Clinical signs were scored as follows: each foot bearing a lesion, 1 point; lesions in or around the mouth, 1 point; and lameness, dullness, or fever (40°C or more), 1 point. Accordingly, the maximum score per animal was 6.

### Virus isolation and titration.

The LFPK-αvβ6 cells were prepared using DMEM supplemented with 10% fetal bovine serum (FBS) in 24-well plates at 2 days before virus isolation. Ten-fold dilutions of the clinical samples were serially prepared in tubes in order to determine the virus titers in the samples. After the cells were washed once, a 100-μl volume of each dilution of the clinical samples was transferred to 4 wells of the 24-well plates and incubated at 37°C for 1 h. The cells were washed again and added to the DMEM supplemented with 10% FBS. The cells were incubated at 37°C for 72 h in 5% CO_2_ and observed microscopically for the appearance of a cytopathic effect (CPE). Virus isolation and titration were performed on the day when each clinical sample was obtained in order to minimize any decrease in virus titers during chilled storage or due to the freezing and thawing processes. Virus titers were calculated according to the Reed-Muench method.

### Virus neutralization test.

A virus neutralization test (VNT) was performed using LFPK-αvβ6 cells as previously described (40). FMDV O/JPN/2000 was used as antigens in the VNT in order to determine antibody responses to the virus in the infected animals.

### Viral growth in cell culture.

BK or LFPK-αvβ6 cell monolayers cultured in 12-well plates were inoculated with the O/JPN/2000 and O/JPN/2010-290/1E strains at a multiplicity of infection (MOI) of 0.1. The culture supernatant was then harvested at subsequent time points and the amount of virus present was determined by virus titration using LFPK-αvβ6 cells. To identify plaque morphology of the two strains, IBRS-2 or ZZR-127 cell monolayers grown in 6-well plates were inoculated and incubated at 37°C for 1 h. The monolayers were then overlaid with MEM containing 0.8% Noble agar and incubated for 1 day at 37°C in 5% CO_2_. Monolayers were fixed with 5% formalin and stained with crystal violet to visualize plaques.

### Nucleotide sequencing.

Viral RNA was extracted from the supernatant of infected cells using a High Pure Viral RNA kit (Roche Diagnostics, Tokyo, Japan), and the L fragment gene, approximately 7.7 kb, was amplified by PCR using four pairs of FMDV-specific primers. The nucleotide sequences were analyzed using the Ion PGM system as previously described (41). The genomes were annotated using GENETYX software with O/JPN/2010-290/1E (GenBank LC036265) as the reference sequence. The locations of the amino acid differences found in the VP1 and 3D between O/JPN/2000 and O/JPN/2010 in the three-dimensional structures were indicated using MOE software (Chemical Computing Group, Montreal, QC, Canada). The amino acid differences were plotted on the three-dimensional structure of VP1 or 3D obtained from the Protein Data Bank (PDB; accession numbers 5NER or 4WZM, respectively).

### Infection of suckling mice.

Animal experiments using suckling mice were performed according to the method described by Platt (16). Two-to-five-day-old BALB/c suckling mice were inoculated intraperitoneally with 100 μl of each serially diluted virus with DMEM. Suckling mice were observed for 1 week after inoculation. LD_50_ was calculated according to the Reed-Muench method.

### Cloning of virus genes and rescue of chimeric viruses.

The 5′ UTR of the L gene, IRES, and the first half and second halves of P1, P2, P3, VP1, 3A-B, 3C, and 3D genes of O/JPN/2000 were amplified by PCR using KOD-Plus-Neo (ToYoBo, Osaka, Japan) and the primers described in Table 3. The 5′ UTR of the L gene, IRES, and the first and second halves of P1, P2, and P3 genes were then ligated with the vectors using appropriate restriction enzymes described in Fig. 3 and a TaKaRa DNA ligation kit ver. 2.1. pSVL-f02, which was constructed in the present study (39), was subjected to PCR to amplify insertion vectors, except for VP1, 3A-B, 3C, and 3D genes, which were constructed using a KOD-Plus mutagenesis kit and the primers described in Table 3 and recombined using an In-Fusion HD Cloning kit (TaKaRa). DNA transfection was performed using Lipofectamine 3000 (Life Technologies) according to the manufacturer’s protocol. Briefly, 2.5 ng of each plasmid was transfected into Cos-7 cells grown to 70% to 90% confluence in each well in a 12-well culture plate. The culture was incubated at 37°C in 5% CO_2_ for 3 days. The supernatants and cells were collected and clarified by low-speed centrifugation at 5,000 × *g* for 10 min after two freeze-thaw cycles. The recovered virus from cells transfected with pSVL-f02 was named vSVL-f02, and other chimeric viruses were named as in Table 3. For chimeric viruses, passage history was aligned with parental virus vSVL-f02 as previously described (39). Namely, the infectious virus from Cos-7 cells was passaged three times in ZZR-127 cells then subsequently in BHK and ZZR-127 cells to obtain a high-titer viral sample for subsequent studies. Genome sequences of all virus stocks of chimeric viruses were confirmed by the method described above.

### Measurement of mutation frequencies.

Virus stocks of O/JPN/2000 and O/JPN/2010 described above were plaque purified and propagated one time in ZZR-127 cells. Viral RNAs were extracted, and part of each P1 structural gene was amplified by PCR using the SuperScript III one-step RT-PCR system with Platinum *Taq* High Fidelity (Life Technologies) and a primer set, 5′-GTGGCATGTAGCGACGGTTA-3′ and 5′-CGTGTTTCACTGCCACCTCTAG-3′. The PCR product was cloned using a TOPO TA Cloning kit (Thermo Fisher) for sequencing. The sequence data were analyzed using GENETYX software. For each population, 70 partial P1 sequences of approximately 700 nt per replicate (genome positions 2699 to 3398 of O/JPN/2010-290/1E) were sequenced. Mutation frequencies per 10^4^ nt were determined as described previously (42).

### Ethics.

The Animal Care and Use Committee of the National Institute of Animal Health (NIAH) approved all animal procedures prior to the initiation of this study (authorization numbers 16-001, 17-066, and 18-038). All experimental infections using live viruses were performed in a high-containment facility at the NIAH.
